# Epitranscriptomic Regulation of Hepatitis B Virus by RNA 5-Methylcytosine: Functions, Mechanisms, and Therapeutic Potential

**DOI:** 10.3390/v17091159

**Published:** 2025-08-24

**Authors:** Xuliu Zhou, Yanling Huang, Xueyan Zhang, Wuxiang Guan, Fang Zhang, Haojie Hao

**Affiliations:** 1 School of Pharmacy, Hubei University of Chinese Medicine, Wuhan 430065, China; 2 Center for Emerging Infectious Diseases, Wuhan Institute of Virology, Chinese Academy of Sciences, Wuhan 430071, China; 3 Hubei JiangXia Laboratory, Wuhan 430200, China

**Keywords:** hepatitis B virus (HBV), 5-methylcytosine (m^5^C), NSUN2, RNA modification, viral replication, hepatocellular carcinoma (HCC)

## Abstract

Hepatitis B virus (HBV) remains a major global health challenge, with over 296 million people chronically infected worldwide. Despite the availability of antiviral therapies, a functional cure is rarely achieved, highlighting the need for novel therapeutic strategies. RNA 5-methylcytosine (m^5^C) is a pivotal epitranscriptomic mark implicated in RNA stability, transport, and translation. Emerging evidence shows that m^5^C is conserved within HBV RNA and plays critical roles in the viral life cycle. This review provides a comprehensive overview of the molecular mechanisms governing m^5^C deposition and recognition, summarizes recent advances in m^5^C biology, and highlights the emerging role of epitranscriptomic m^5^C regulation in HBV infection. We discuss the identification of HBV-specific m^5^C sites, the functions of key regulatory enzymes, and their interplay in viral RNA stabilization and evasion of innate immune responses. Interplay between m^5^C and other RNA modifications—particularly N6-methyladenosine (m^6^A)—is examined alongside virus-specific m^5^C regulation in EV71, HIV, HCV, EBV, and SARS-CoV-2. Potential links between m^5^C dysregulation and HBV-induced hepatocarcinogenesis are outlined, and emerging therapeutic strategies targeting the m^5^C machinery are highlighted. Together, these insights position the epitranscriptomic landscape as a promising avenue for innovative antiviral strategies.

## 1. Introduction

Hepatitis B virus (HBV) causes chronic infections in over 290 million individuals worldwide and remains a leading cause of liver cirrhosis and hepatocellular carcinoma (HCC) [1]. Despite the availability of effective vaccines and antiviral agents, complete viral eradication remains elusive due to the persistence of viral RNA species and the stable reservoir of infected hepatocytes [1]. HBV is an enveloped, hepatotropic virus with a partially double-stranded, circular genome of approximately 3.2 kb [2]. Its replication relies on reverse transcription of a pregenomic RNA (pgRNA) intermediate, ultimately generating covalently closed circular DNA (cccDNA), which serves as a stable transcriptional template and underlies viral persistence [3].

In recent years, post-transcriptional regulation of viral and host RNAs via epitranscriptomic modifications has emerged as a crucial layer of host–virus interaction. To date, more than 180 distinct chemical modifications have been identified on RNA [4], with the most extensively studied including N6-methyladenosine (m^6^A), 5-methylcytosine (m^5^C), pseudouridine (Ψ), N7-methylguanosine (m^7^G), N1-methyladenosine (m^1^A), 2′-O-Methylation (Nm), and N^4^-acetylcytidine (ac4C) [5]. These modifications play important roles in shaping both host cell and viral processes during infection (Table 1). Among these, m^5^C—methylation at the fifth carbon of cytosine—is second only to m^6^A in terms of research prominence. It has been implicated in diverse RNA regulatory processes, including RNA stability [6,7], translation [8], selective cleavage, and nuclear export [9].

Despite growing interest in RNA modifications during viral infections, the role of m^5^C in HBV infection remains poorly defined. The molecular mechanisms governing m^5^C deposition and its functional consequences in the context of HBV replication, immune evasion, and pathogenesis have yet to be fully elucidated. This represents a critical gap in our understanding of the HBV life cycle and its interplay with host RNA metabolism. Addressing this knowledge gap may uncover novel therapeutic targets and enhance our comprehension of the epitranscriptomic dimension of virus–host interactions.

## 2. RNA m^5^C: Distribution and Regulatory Mechanisms

m^5^C is a widespread and conserved modification found across multiple RNA species, including messenger RNAs (mRNAs), transfer RNAs (tRNAs), ribosomal RNAs (rRNAs), and other noncoding RNAs (ncRNAs) [38]. Over 10,000 potential m^5^C modification sites have been detected throughout the human transcriptome [39]. In eukaryotic cells, m^5^C sites are enriched in untranslated regions, coding sequences, and near splice sites, with differential distribution between nuclear and cytoplasmic transcripts [40]. As an important form of post-transcriptional modification of RNA, m^5^C widely affects gene expression and biological processes at multiple levels.

### 2.1. m^5^C Writers

**Figure 1:**
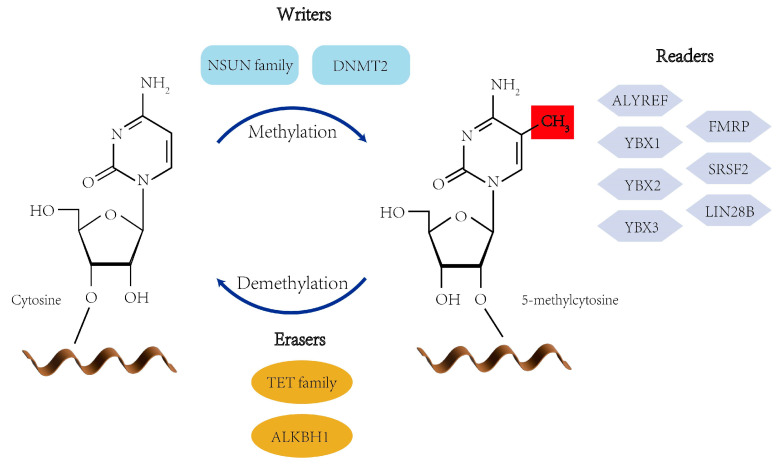
Writer, eraser, and reader proteins of RNA m^5^C modification. Writers of RNA m^5^C modification include NSUN family and DNMT2, erasers of RNA m^5^C modification include TET family and ALKBH1, and readers of RNA m^5^C modification include YBX1-3, ALYREF, FMRP, SRSF2, and LIN28B.

Methyltransferases (writers) transfer a methyl group from S-adenosyl-L-methionine (SAM) to the C5 position of cytosine to form 5-methylcytosine [41]. The human genome encodes at least eight catalytically active m^5^C writers, comprising DNA methyltransferases 2 (DNMT2, also known as TRDMT1) and members of the NOP2/Sun (NSUN) family (NSUN1–7). Unlike canonical DNMTs (DNMT1, DNMT3A, and DNMT3B), DNMT2 primarily targets RNA, methylating cytosine 38 of specific tRNAs [42], and has also been implicated in mRNA methylation [43,44]. The NSUN family members exhibit substrate and subcellular specificity: NSUN1 (NOP2) and NSUN5 methylate rRNAs in the nucleolus, NSUN3 and NSUN4 operate in mitochondria to modify mitochondrial tRNAs (mt-tRNAs) and mt-rRNAs, and NSUN6 targets cytoplasmic tRNAs [45], whereas NSUN2—predominantly localized in the nucleus but also present in the cytoplasm—is responsible for m^5^C modification on a broad spectrum of substrates, including rRNAs [46], tRNAs [47], mRNAs [48], mt-RNAs [49], and tsRNAs [50]. Notably, several m^5^C writers—NSUN1, NSUN2, NSUN5, and DNMT2—have been shown to methylate viral RNAs [51], highlighting conserved host–virus enzymatic machinery that shapes the epitranscriptomic landscape during infection. This convergence suggests that viruses may co-opt host RNA modification systems to fine-tune their gene expression, enhance replication efficiency, and evade host immune surveillance (Figure 1).

### 2.2. m^5^C Erasers

The removal of 5-methylcytosine (m^5^C) marks on RNA is catalyzed by specific demethylases (erasers), including ten-eleven translocation (TET) family dioxygenases (TET1–3) and the Fe(II)/α-ketoglutarate-dependent enzyme ALKBH1 [52]. The TET enzymes oxidize m^5^C to 5-hydroxymethylcytosine (hm^5^C), a key intermediate in active demethylation. Among them, TET2 has been identified as the first RNA m^5^C demethylase, with a particularly important role in regulating dynamic m^5^C turnover on mRNAs during hematopoietic differentiation and cellular stress responses [53,54,55]. In contrast, ALKBH1 exhibits substrate specificity for tRNAs, where it demethylates m^5^C through a stepwise oxidation to hm^5^C and subsequently to 5-formylcytosine (f5C) [56]. The resulting f5C can pair with adenine, thereby reshaping RNA secondary structure [57]. ALKBH1 activity is critical for maintaining tRNA structure and decoding fidelity, particularly under metabolic or oxidative stress conditions. Together, TETs and ALKBH1 confer reversibility to m^5^C modifications, enabling cells to dynamically remodel the epitranscriptomic landscape in response to environmental cues, developmental signals, or pathogenic stress. Their involvement in viral RNA regulation is only beginning to be explored, raising the possibility that viruses may exploit these host erasers to modulate RNA fate and immune recognition (Figure 1).

### 2.3. m^5^C Readers

Reader proteins specifically recognize m^5^C-modified RNAs and regulate their functional outcomes by controlling RNA export, stability, translational efficiency, and subcellular localization. In eukaryotic cells, the currently identified m^5^C readers include ALYREF, YBX1, YBX2, YBX3, and FMRP (Fragile X Mental Retardation Protein) [52,58,59,60]. Among them, ALYREF recognizes m^5^C via lysine 171 (K171) and promotes the nuclear export of modified mRNAs [61]. Its recruitment is initiated at the 5′ end of the transcript via CBP80, followed by association with the 3′ end through interactions with PABPN1 and further stabilization via the 3′ end processing factor CstF64 [62]. YBX1 binds m^5^C through the tryptophan 65 (W65) within its cold-shock domain, stabilizing transcripts in the cytoplasm [58]. YBX2 similarly engages m^5^C via W100 of the cold-shock domain to facilitate liquid–liquid phase separation in germ cells [58]. Zhang et al. demonstrated that NSUN6-mediated m^5^C sites recruit both YBX1 and YBX3, thereby increasing mRNA stability [60]. FMRP has also been implicated in the m^5^C pathway through its interaction with TET1, where it facilitates R-loop demethylation and indirectly links m^5^C dynamics to DNA damage repair [59]. In addition, emerging evidence suggests that other RNA-binding proteins, such as SRSF2 and LIN28B, may act as m^5^C readers, expanding the functional repertoire of this modification to include alternative splicing regulation, translational control, and developmental timing [63,64] (Figure 1). These findings underscore the diverse and context-dependent interpretation of m^5^C marks by specialized reader proteins.

## 3. Biological Functions of m^5^C Methylation

The biological roles of m^5^C are increasingly recognized in both coding and ncRNAs. Like m^6^A, m^5^C modifications regulate RNA metabolism by modulating mRNA translation, stability, and nuclear export, as well as maintaining the structural integrity and function of tRNAs and rRNAs [65].

### 3.1. m^5^C in mRNAs

**Figure 2:**
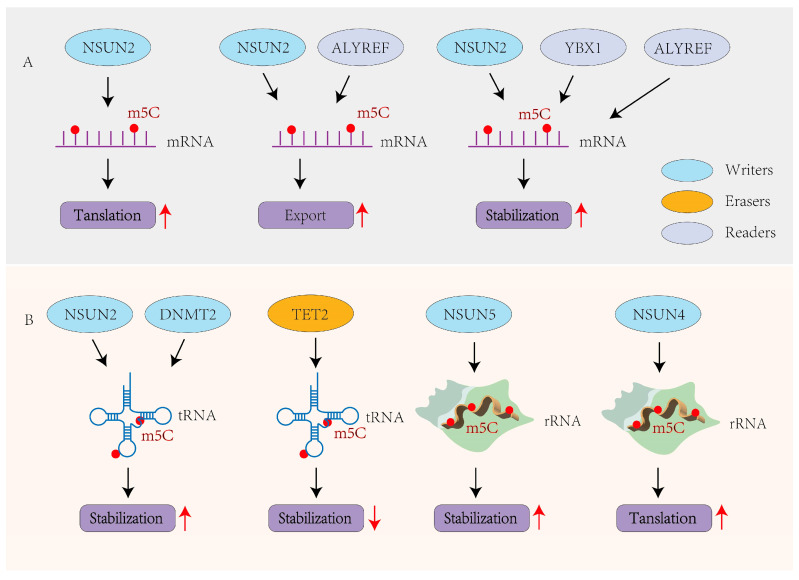
Biological function of m^5^C methylation modification. (**A**) m^5^C modification regulates mRNA translation, nuclear export, and stability. (**B**) m^5^C modification enhances ncRNA stability and translation.

m^5^C primarily affects mRNA fate through three mechanisms: translation, nuclear export, and stability. Modifications within coding sequences are generally associated with reduced translation efficiency, while those in 3′ UTRs typically enhance translation [66,67]. NSUN2-catalyzed m^5^C modifications are recognized by ALYREF, which facilitates mRNA export from the nucleus to the cytoplasm [61,68]. Additionally, m^5^C enhances mRNA stability by promoting interactions with proteins like YBX1, contributing to oncogenic gene expression in cancers such as bladder and cervical cancer [6,69]. Two other parallel studies in humans and zebrafish have also shown that YBX1 regulates m^5^C-dependent mRNA stability [6,70]. Moreover, ALYREF influences the stability and splicing of mRNAs by binding to the m^5^C-modified sites on transcripts such as PKM2 [71], RABL6, and TK1 [72] (Figure 2A). In cancer contexts, ALYREF enhances BIRC5 mRNA stability via m^5^C, driving ovarian tumor progression [73], and upregulates NOTCH1 in an m^5^C-dependent manner to activate NOTCH signaling and facilitate nasopharyngeal carcinoma metastasis [74].

### 3.2. m^5^C in ncRNAs

m^5^C modifications in ncRNAs, especially in tRNAs and rRNAs, play key roles in RNA stability and translational control [52,54] (Figure 2B). In tRNAs, m^5^C is mainly found in the variable region and anticodon loop, where it stabilizes the RNA structure and prevents degradation [75]. In humans, mice, and plants, m^5^C methylation mediated by TRM4/NSUN2 or DNMT2 protects specific tRNAs such as tRNA^Asp-GTC^ and tRNA^Gly-GCC^ from oxidative cleavage or exonuclease activity [66,76]. TET2 has also been shown to modulate tRNA function by oxidizing m^5^C, linking m^5^C dynamics to translational regulation [54]. In rRNA, NSUN5 modifies 25S rRNA at C2278, and its loss under oxidative stress disrupts local folding and ribosome stability [77]. NSUN4 methylates 12S mitochondrial rRNA, supporting mitoribosome assembly and function [78]. Furthermore, m^5^C sites have been identified in lncRNAs, where they are thought to influence RNA processing and stability, protein translation, and RNA–protein interactions, though their precise functions remain under investigation [79,80].

## 4. Detection Methods of RNA m^5^C Methylation

Although m^5^C was first identified in RNA as early as the 1970s [81,82], the limited sensitivity and resolution of early technologies restricted its detection, particularly in structured or low-abundance RNAs, thereby impeding transcriptome-wide profiling and precise site-specific characterization. In recent years, rapid advances in high-throughput sequencing, mass spectrometry, and chemical biology have enabled more precise and comprehensive detection of RNA m^5^C modifications. Current techniques vary in terms of resolution, specificity, throughput, and RNA class compatibility, and often require integrative approaches to yield accurate biological insights.

### 4.1. RNA Bisulfite Sequencing (RNA-BisSeq)

**Figure 3:**
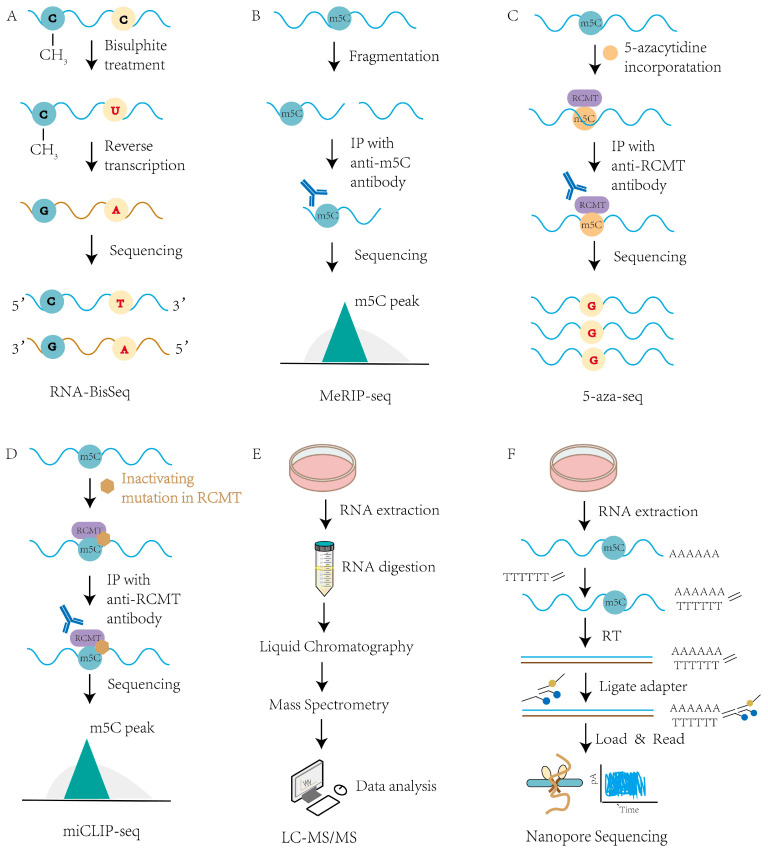
Detection methods of RNA m^5^C methylation. The detection methods for RNA m^5^C methylation mainly include RNA-BisSeq (**A**), MeRIP-seq (**B**), 5-aza-seq (**C**), miCLIP-seq (**D**), LC-MS/MS (**E**) and nanopore sequencing (**F**).

RNA-BisSeq, originally developed for DNA methylation analysis [83], was later adapted to RNA [84] and remains one of the few methods capable of mapping m^5^C at single-nucleotide resolution. This approach relies on bisulfite treatment, which deaminates unmethylated cytosines into uracil while preserving m^5^C intact. Subsequent reverse transcription and sequencing then enable precise mapping of methylated sites (Figure 3A). Despite its specificity, RNA-BisSeq has key limitations: it requires large amounts of high-quality RNA, is sensitive to degradation due to harsh chemical conditions, and is less reliable in structured or low-abundance transcripts [85]. Furthermore, incomplete conversion and mapping ambiguities can generate false positives or negatives, especially in regions with strong secondary structures.

### 4.2. Immunoprecipitation-Based Techniques

Antibody-based methods such as MeRIP-seq enrich for m^5^C-modified fragments and offer transcriptome-wide coverage [86] (Figure 3B). However, this method suffers from low resolution and is dependent on antibody specificity and affinity, limiting its ability to precisely localize individual m^5^C sites [86]. A more refined chemical approach, 5-azacytidine-mediated RNA immunoprecipitation (5-aza-IP), takes advantage of the covalent trapping of methyltransferase–RNA complexes using 5-azacytidine, enabling detection of enzyme-specific m^5^C sites, such as those deposited by NSUN2 or DNMT2 [87] (Figure 3C). Nevertheless, the cytotoxicity and nonspecific incorporation of 5-aza analogs pose significant challenges. An alternative approach, miCLIP (methylation individual-nucleotide resolution crosslinking and immunoprecipitation), exploits catalytically dead mutants of RNA cytosine methyltransferases (RCMTs) that form an irreversible covalent bond with their m^5^C substrates. Following partial RNA fragmentation, these RCMT–RNA adducts are enriched by anti-RCMT immunoprecipitation, and library preparation captures the characteristic reverse-transcription stops or misincorporations at the crosslinked site. Sequencing and alignment thus yield single-nucleotide resolution maps of m^5^C positions [88] (Figure 3D). While highly specific, miCLIP is technically complex and often requires overexpression systems, which may introduce artifacts.

### 4.3. LC-MS/MS and Nanopore Sequencing

LC-MS/MS (Liquid Chromatography–Tandem Mass Spectrometry) enables quantitative detection of global m^5^C levels across RNA populations by separating and detecting modified nucleosides (Figure 3E) but lacks positional information and cannot distinguish between RNA species [85]. To address this, third-generation sequencing platforms, such as single-molecule real-time (SMRT) sequencing and nanopore-based direct RNA sequencing, have emerged as powerful tools for native RNA modification detection [89]. These technologies can detect changes in electrical signals or pulse kinetics induced by nucleotide modifications in real time, without the need for chemical conversion or amplification (Figure 3F). Notably, nanopore sequencing enables full-length RNA molecule detection and isoform-specific modification profiling. Although current limitations include elevated error rates, underdeveloped modification-calling algorithms, and challenges in discriminating similar epitranscriptomic marks, this technology provides distinct advantages—particularly when integrated with conventional biochemical approaches in comprehensive analytical pipelines.

## 5. Molecular Mechanisms of HBV-Related HCC

Hepatocellular carcinoma (HCC) is the most frequent primary liver cancer, with chronic HBV infection as a principal etiological factor [90]. HBV induces hepatocarcinogenesis through complex mechanisms involving both direct viral oncogenic effects and virus-mediated alterations of host cell processes [74]. Central to this process are the persistence of covalently cccDNA, integration of HBV DNA into the host genome, accumulation of viral mutations, and the pleiotropic activities of viral proteins—particularly the hepatitis B virus X protein (HBx) [91]. In addition, growing evidence highlights the contribution of epigenetic dysregulation, including DNA methylation and histone modifications, as well as immune evasion and chronic inflammation, in HBV-driven hepatocarcinogenesis [8,9,92].

### 5.1. HBV Replication Cycle

**Figure 4:**
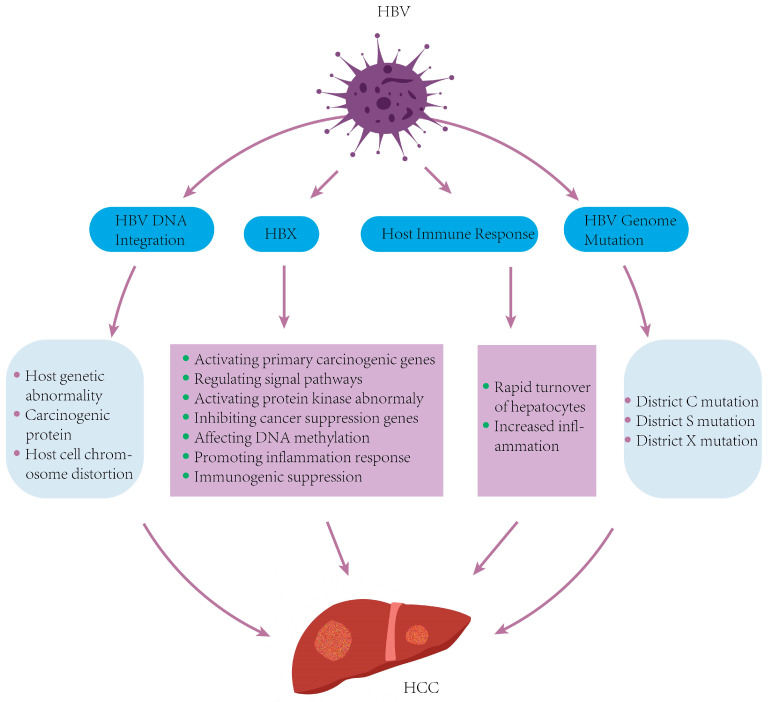
Mechanisms of HBV-induced HCC formation. The molecular mechanisms of HBV-related HCC mainly include four aspects: HBV DNA integrates into the host genome, the rapid turnover of hepatocytes and increased inflammation caused by the host immune response, HBV genomic mutations, and abnormal expressions of HBx.

HBV enters host hepatocytes via sodium taurocholate co-transporting polypeptide (NTCP)-mediated endocytosis [93]. Upon entry, the nucleocapsid is released into the cytoplasm and subsequently transported into the nucleus, where it releases the relaxed circular DNA (rcDNA). The host DNA repair machinery then converts rcDNA into episomal covalently cccDNA, which serves as the transcriptional template for viral RNAs, including pgRNA (encoding core protein and viral polymerase) and subgenomic RNAs (encoding envelope proteins and HBx) [94]. The pgRNA–polymerase complex is encapsidated, followed by reverse transcription to synthesize rcDNA. Mature nucleocapsids either recycle to the nucleus to replenish the cccDNA pool or acquire envelope proteins (HBsAg) in the endoplasmic reticulum to form complete virions for secretion. Notably, the persistence of cccDNA and the oncogenic activity of HBx protein constitute key mechanisms underlying HBV-related HCC pathogenesis [95] (Figure 4).

### 5.2. HBV DNA Integration into the Host Genome

Integration of HBV DNA into the host genome is a hallmark of chronic infection and is detected in up to 80–90% of HBV-related HCC cases [95]. This process is primarily mediated by host DNA double-strand break repair pathways, including non-homologous end joining (NHEJ) and microhomology-mediated end joining (MMEJ), and typically occurs shortly after infection or during hepatocyte proliferation, when genomic instability and DNA damage are more prevalent [96]. Integrated HBV DNA tends to insert into vulnerable genomic regions, such as CpG islands, telomeric zones, and cancer-associated loci, such as TERT, MLL4, and CCNE1 [97]. These events can lead to insertional mutagenesis, activation of proto-oncogenes, disruption of tumor suppressor genes, and induction of chromosomal instability. In addition to altering gene expression, integrated HBV DNA can generate truncated viral transcripts and chimeric fusion proteins with oncogenic potential [98]. Importantly, integrated HBV DNA is replication-defective and does not contribute to the production of infectious virions, but it continues to express viral proteins—particularly HBx and HBsAg—that can chronically stimulate the immune system, promote immune escape, and contribute to tumor microenvironment remodeling [99]. Such changes may act independently or in synergy to promote tumorigenesis. Current antiviral therapies fail to eliminate integrated HBV DNA or cccDNA, making these elements persistent drivers of liver disease. Therefore, understanding the mechanisms and consequences of HBV DNA integration is essential for guiding efforts to prevent or delay disease progression (Figure 4).

### 5.3. HBV Genomic Mutations

HBV is a DNA virus with a high mutation rate due to the lack of proofreading activity by its reverse transcriptase. The viral genome includes four open reading frames (ORFs)—S, P, C, and X [100], with mutations in the C, S, and X regions linked to HCC, while those in the P region often relate to antiviral resistance [101]. For instance, the G1896A mutation in the pre-C region generates a premature stop codon in the hepatitis B e antigen (HBeAg) ORF, reducing its expression without hindering viral replication. This leads to persistent infection, exacerbating liver injury and promoting cancer progression [102]. Mutations in the pre-S region can cause the intracellular accumulation of surface proteins in the endoplasmic reticulum (ER), inducing ER stress, oxidative stress, and DNA damage response pathways, which ultimately drive tumor formation. Point mutations, deletions, or insertions in the pre-S sequences further increase HCC risk [103]. Additionally, HBx, encoded by the X region, plays a crucial role in hepatocyte transformation [104]. Mutations like T1753V and A1762T/G1764A in the core promoter region are frequently associated with HCC and may promote tumor progression through altered regulation of genes such as TGF-β1 [105,106] (Figure 4).

### 5.4. Abnormal Expression of the HBx Gene and Its Encoded Protein

The HBx gene, one of four overlapping ORFs in the HBV genome, encodes the multifunctional HBx protein, a key regulator in HBV pathogenesis [107]. Certain mutations—particularly K130M and V131I—are associated with a 4- to 5-fold increased risk of HCC [108]. HBx can interact directly with host transcription factors and activate intracellular signaling cascades [107]. It promotes HCC development by activating proto-oncogenes such as RAS, C-FOS, and C-MYC, driving cell proliferation and differentiation [109]. HBx also modulates pathways like JAK-STAT, Notch, and MAPK, enhancing cancer progression [110,111,112]. Additionally, HBx contributes to tumorigenesis by inhibiting tumor suppressors like p53 [113], altering DNA methylation [114], inducing inflammation [115], and suppressing host immune responses [105] (Figure 4).

## 6. The Role of m^5^C in the HBV Life Cycle

Epitranscriptomic modifications regulate critical phases of viral infection. In HBV, the pgRNA functions dually as the reverse transcription template and as the bicistronic mRNA encoding core and polymerase proteins. While m^6^A modifications on pgRNA are known to enhance encapsidation, reverse transcription, and immune evasion [116,117,118], m^5^C has emerged as another pivotal regulator of HBV replication and host interactions.

### 6.1. m^5^C Enhances pgRNA Stability and Encapsidation

**Figure 5:**
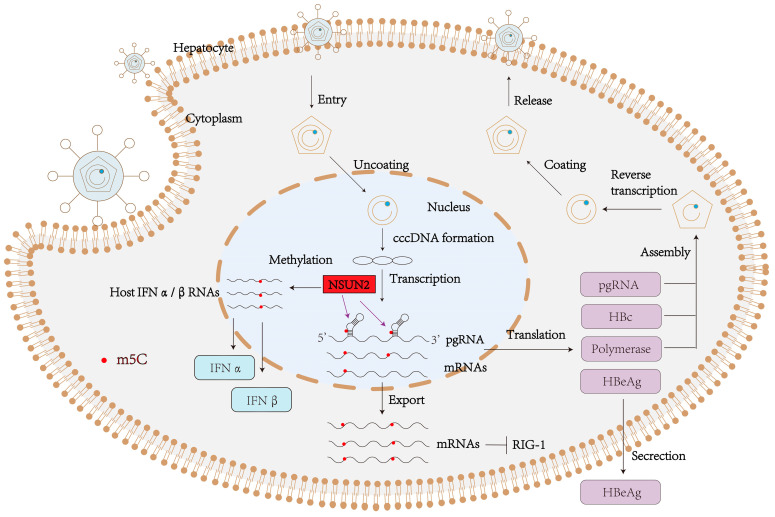
The role of m^5^C in the HBV life cycle. NSUN2-mediated m^5^C methylation on pgRNA enhances its stability and promotes specific recognition by the viral core protein (HBc), facilitating efficient nucleocapsid assembly. This modification serves three key functions: (1) it strengthens pgRNA-HBc interactions through structural remodeling of the 5′ ε stem-loop, (2) enhances reverse transcription efficiency by maintaining optimal RNA conformation for polymerase binding, and (3) mediates immune evasion through dual mechanisms—impairing RIG-I recognition of viral RNA while suppressing HBc-induced inflammatory responses. The NSUN2-dependent m^5^C modification thus coordinates multiple stages of HBV replication while simultaneously counteracting host antiviral defenses.

m^5^C modification plays a key role in maintaining the stability of HBV pgRNA, the essential intermediate responsible for both protein translation and the reverse transcription of the viral genome. High-resolution bisulfite sequencing has identified multiple m^5^C-modified cytosines within the pgRNA, particularly at C131 in the ε element and C2017 in the 3′ UTR [119]. These sites are evolutionarily conserved and are catalyzed by NSUN2 [119]. Loss of NSUN2, via genetic knockdown or knockout, significantly diminishes HBV RNA levels, viral DNA synthesis, and secretion of HBeAg. Furthermore, site-directed mutagenesis of C131 or C2017 to uracil impairs RNA stability, underscoring the functional importance of these modifications [119]. Mechanistically, m^5^C appears to enhance the association between pgRNA and viral core proteins, facilitating efficient encapsidation [120]. Stability assessments further show that m^5^C-containing pgRNAs exhibit a longer half-life compared to unmethylated variants, supporting the role of m^5^C in protecting viral RNA from degradation (Figure 5).

### 6.2. m^5^C in Element (ε) Regulates HBV Reverse Transcription

Reverse transcription of pgRNA into HBV DNA is initiated at the ε stem-loop structure located at the 5′ end of the pgRNA. This structure is required for viral polymerase recognition and serves as the initiation site for (-)-strand DNA synthesis. Several cytosines in ε are subject to m^5^C modification in an NSUN2-dependent manner. Disruption of ε methylation, either by mutating the cytosines or NSUN2 depletion, abolishes reverse transcription, leading to the formation of non-infectious empty capsids. Reintroducing m^5^C via NSUN2 restoration rescues DNA synthesis and mature virion production, demonstrating that m^5^C marks act as essential molecular cues for effective reverse transcription and virion maturation [120]. Moreover, m^5^C marks in ε are functionally complementary to m^6^A marks in the same region [120], suggesting that a complex epitranscriptomic network regulates the RNA structural landscape to orchestrate viral life-cycle events (Figure 5).

### 6.3. m^5^C Modulates Innate Immune Recognition and Viral Evasion

Unlike many DNA viruses, HBV primarily triggers host innate immunity through RNA-sensing mechanisms rather than DNA-sensing pathways. This is largely because hepatocytes express low levels of key DNA-sensing factors, such as the stimulator of interferon genes (STING), rendering RNA pattern recognition receptors—particularly retinoic acid-inducible gene I (RIG-I)—the principal sensors of HBV infection [121]. Our recent studies have revealed that m^5^C methylation of HBV RNA plays a critical role not only in promoting viral gene expression but also in helping HBV evade host innate immunity [122]. Specifically, we identified a conserved m^5^C site at nucleotide 1291 on the HBV transcript, which serves dual functions in regulating viral mRNA export and interfering with immune sensing. Mechanistically, m^5^C at nt1291 facilitates the recruitment of the nuclear export adaptor ALYREF, thereby promoting the efficient cytoplasmic export of HBV mRNA. This enhances the translation of viral proteins, notably HBx, and contributes to productive viral replication. Loss of this methylation mark—either through NSUN2 knockdown or site-directed mutation—disrupts ALYREF binding, impairs HBx translation, and reduces viral propagation. More importantly, m^5^C at nt1291 also plays an essential role in antagonizing the host RNA sensor RIG-I. We demonstrated that methylation at this site inhibits the binding of RIG-I to HBV RNA, thereby preventing the downstream activation of the mitochondrial antiviral-signaling protein (MAVS) pathway and blocking interferon-β (IFN-β) production. In the absence of m^5^C, RIG-I recognition is restored, leading to a significant upregulation of IFN-β and reactivation of the host antiviral response. In addition to modifying its own RNA, HBV also actively modulates host m^5^C epitranscriptomic machinery. We found that HBV infection downregulates the m^5^C methyltransferase NSUN2 in hepatocytes, leading to reduced m^5^C methylation on host interferon-related host mRNAs such as interferon beta 1 (IFNB1). This reduction compromises the stability and expression of these transcripts, thereby attenuating type I interferon production and facilitating viral persistence. Collectively, these findings reveal a dual role for NSUN2-mediated m^5^C methylation in HBV pathogenesis: enhancing viral mRNA export and translation while simultaneously dampening host antiviral signaling. Through this epitranscriptomic strategy, HBV coordinates the manipulation of both viral and host transcriptomes, highlighting m^5^C as a promising therapeutic target in chronic hepatitis B (Figure 5).

## 7. m^5^C Methylation in HBV-Related HCC and Associated Liver Diseases

m^5^C plays a critical role in the initiation and progression of various tumors. Transcriptome-wide analyses reveal distinct m^5^C patterns in HCC versus adjacent non-tumor liver, suggesting a malignancy-specific epitranscriptomic signature [123,124]. One study revealed that the m^5^C reader ALYREF modulates methylation of key transcripts to promote HCC progression [125]. Subsequent research showed that ALYREF binds m^5^C sites on EGFR mRNA, enhancing transcript stability and activating downstream STAT3 signaling to further drive tumor growth [126]. Notably, Sun et al. reported that NSUN2 deposits m^5^C on the lncRNA H19, increasing its stability; m^5^C-modified H19 then recruits the oncoprotein G3BP1, leading to MYC accumulation and poor differentiation in HBV-related HCC [127].

Beyond cancer, m^5^C also modulates other HBV-related liver pathologies. In fibrogenesis, YBX1 knockout in hepatic progenitor cells reduces hepatic stellate cell activation and mitigates liver fibrosis in mice [128], suggesting that m^5^C regulators may influence HBV-associated fibrotic processes. To date, however, there are no direct studies reporting m^5^C regulation in HBV-related hepatitis, cirrhosis, or liver failure. Nevertheless, evidence from other liver disease contexts highlights broader roles of m^5^C. For instance, TET2 modulates alcohol-induced liver injury by regulating Srebp1 mRNA stability [129], and ALYREF cooperates with NSUN2 to promote m^5^C-dependent export and translation of CDKN1A mRNA during adipogenesis in non-alcoholic fatty liver disease [130]. These findings, while not directly HBV-related, provide valuable clues suggesting that epitranscriptomic dysregulation of m^5^C may broadly contribute to liver disease progression and thus warrants further investigation in HBV-associated settings.

Currently, the role of RNA m^5^C modification in HCC is becoming increasingly well-defined, making methylation-related proteins promising therapeutic targets for HCC treatment. Sorafenib, a targeted therapy for advanced HCC, has limited efficacy due to drug resistance. However, combining sorafenib with NSUN1 siRNA significantly enhances HCC sensitivity to sorafenib [131]. Qu et al. identified that targeting the ALKBH5 surface represents a promising therapeutic strategy for HBV-induced HCC [132]. As our understanding of m^5^C regulators in HBV-driven liver disease deepens, targeting writer, eraser, or reader proteins may yield novel antiviral and anti-tumor strategies.

## 8. Crosstalk Between m^5^C and Other Modifications in the HBV Life Cycle

RNA modifications often coexist on the same transcript and cooperate to form an intricate “epitranscriptomic code.” In HBV, the most abundant internal modification, m^6^A, is enriched at the ε packaging signal and within the HBx ORF, where it stabilizes pgRNA, enhances encapsidation, and suppresses RIG-I-mediated immune sensing via YTHDF readers; additionally, m^6^A modification of host PTEN mRNA promotes HBx-mediated immune evasion and hepatocarcinogenesis [133]. Beyond m^6^A, other modifications also contribute to viral RNA regulation: ac4C boosts internal ribosome entry site (IRES)-driven translation in enteroviruses, while Ψ stabilizes RNA secondary structures and improves translational efficiency in various viral contexts.

In the case of HBV, it remains unclear whether m^6^A, ac4C, and Ψ co-localize with m^5^C on the same transcripts, or whether they exert synergistic or antagonistic effects. Elucidating such interactions requires high-resolution mapping of RNA modifications. Emerging technologies such as nanopore direct RNA sequencing and crosslinking-based methods like miCLIP offer promising tools to uncover combinatorial modification patterns. These approaches could help delineate how m^5^C cooperates or competes with other modifications to regulate viral RNA stability, nuclear export, immune evasion, and replication. Hepatitis D virus (HDV), a satellite virus of HBV that significantly exacerbates liver disease pathogenesis, is known to harbor epitranscriptomic modifications such as m^6^A and A-to-I RNA editing. However, whether HDV RNA contains m^5^C modifications remains unexplored, presenting an important avenue for future research [134,135].

## 9. Comparative Insights: m^5^C in Other RNA and DNA Viruses

m^5^C is a widespread and conserved modification found across diverse RNA viruses, with virus-specific functions and regulatory contexts. In enterovirus 71 (EV71), NSUN2-mediated m^5^C installation within the 5′ UTR enhances RNA stability and facilitates IRES-dependent translation [11]. In human immunodeficiency virus type 1 (HIV-1), the genomic RNA is heavily methylated (approximately 11–14 m^5^C-modified sites), which promotes the export of unspliced transcripts, ribosome engagement, and alternative splicing regulation [136]. For severe acute respiratory syndrome coronavirus 2 (SARS-CoV-2), m^5^C marks are distributed across the viral genome in an NSUN2-dependent manner; NSUN2 knockout leads to global hypomethylation of viral RNA, resulting in increased RNA stability, elevated viral loads, and aggravated lung pathology in infected mice [137]. MLV RNAs bear an exceptionally high level of m^5^C epitranscriptomic modifications, and downregulation of the m^5^C writer NSUN2 inhibits MLV replication [138]. In hepatitis C virus (HCV), m^5^C modification within the NS5A coding region enhances RNA stability and replication efficiency, with YBX1 acting as a critical reader protein that mediates these effects [139]. Additionally, a growing body of evidence suggests that other RNA viruses—including Dengue virus, Zika virus, respiratory syncytial virus (RSV), and vesicular stomatitis virus (VSV)—also exploit NSUN2-dependent m^5^C modifications to modulate viral replication and evade host immune responses, although the specific methylation sites and interacting proteins vary across viruses [140,141]. Collectively, these findings highlight both the evolutionary conservation and the functional divergence of m^5^C regulation in RNA viruses. While NSUN2 is a common methyltransferase utilized across pathogens, the biological consequences of m^5^C—ranging from enhanced translation and RNA stability to immune modulation and RNA processing—are highly virus- and context-specific. These comparative insights further underscore the distinct and multifaceted role of m^5^C in HBV pathogenesis and its potential as a therapeutic target (Table 2).

## 10. Clinical and Therapeutic Perspectives

### 10.1. Genotype- and Host-Dependent Variation in m^5^C

HBV comprises at least ten major genotypes (A–J), which differ in their geographic prevalence, disease progression, and treatment response. It remains to be determined whether differential HBV genotypes harbor distinct m^5^C landscapes or NSUN2 affinities. Genotype-specific pgRNA secondary structures may influence the accessibility of m^5^C writers or readers, potentially affecting viral replication efficiency and immune recognition. Likewise, vaccine non-responders may possess altered epitranscriptomic profiles that compromise viral antigen expression, while antiviral-resistant strains may harbor polymerase mutations near m^5^C sites, potentially interfering with methylation or disrupting reader recruitment. Comparative methylome profiling across diverse HBV genotypes and patient cohorts will be essential to elucidate these genotype- and host-specific variations and their implications for disease progression and therapeutic response.

### 10.2. m^5^C in HBV-Driven Hepatocarcinogenesis

Dysregulation of the m^5^C machinery has been implicated in hepatocarcinogenesis. In HCC, NSUN2 is frequently overexpressed and associated with poor prognosis, enhanced cellular proliferation, and impaired antiviral defense [144]. As an m^5^C reader, ALYREF stabilizes oncogenic transcripts—such as EGFP—thereby promoting downstream signaling pathways like STAT3, which are known to drive tumor progression [126]. Moreover, persistent NSUN2-mediated methylation of host immune-related mRNAs, such as TREX2 and IRF3, may undermine DNA sensing and interferon responses, creating an immunosuppressive microenvironment that favors malignant transformation [140]. In the setting of chronic HBV infection, sustained m^5^C activity may therefore facilitate malignant transformation by both subverting innate immunity and promoting proliferative signaling.

### 10.3. Targeting the m^5^C Machinery for Therapy

Given the dual roles of m^5^C in viral persistence and tumorigenesis, the RNA methylation machinery presents an attractive therapeutic target. Small-molecule inhibitors of NSUN2, or agents that disrupt ALYREF–RNA interactions, may destabilize HBV pgRNA and restore immune sensing. Conversely, enhancing the activity of demethylases such as TET2 might promote the removal of m^5^C from viral transcripts, accelerating their degradation. Cutting-edge technologies—such as dCas13-based epitranscriptomic editors—could be deployed to selectively erase m^5^C marks at specific HBV RNA loci. Finally, combining m^5^C-targeted strategies with standard-of-care nucleoside analogs or immunotherapies may yield synergistic effects, suppressing both viral replication and HCC progression. Nonetheless, several challenges must be addressed—chief among them the need for high specificity toward viral versus host transcripts, adequate delivery across hepatocyte membranes, and minimal off-target effects. Future work should focus on developing selective m^5^C modulators and validating their efficacy in relevant in vivo models.

## 11. Conclusions and Outlook

RNA m^5^C has emerged as a pivotal epitranscriptomic mark that governs RNA stability, processing, localization, and immune recognition in both eukaryotic cells and a growing array of RNA viruses. In the context of HBV, m^5^C—catalyzed primarily by the methyltransferase NSUN2—plays a multifaceted role in viral replication and host–pathogen interactions. Specific m^5^C modifications at conserved loci on the pgRNA, including C131, multiple residues within the ε encapsidation signal, C2017 in the 3′ UTR, and nt1291, contribute to enhanced RNA stability, efficient reverse transcription, and evasion of innate immune sensing. These effects are mediated in part through the recruitment of m^5^C readers such as ALYREF, which facilitate viral mRNA export and translational efficiency. Intriguingly, HBV also actively suppresses NSUN2 expression in infected hepatocytes, leading to reduced m^5^C methylation on host interferon-related transcripts such as IFNB1, thereby dampening type I interferon responses and promoting viral persistence.

Despite these advances, several challenges and opportunities remain. First, the interplay among m^5^C writers, erasers, and readers has yet to be mapped comprehensively in HBV and host transcripts; elucidating these regulatory networks and how they interact with other RNA modifications, such as m^6^A, ac4C, and Ψ, is essential for decoding the “epitranscriptomic code” that governs viral RNA fate. Second, genotype-dependent differences in HBV RNA structure may influence m^5^C deposition and function—a question that demands comparative methylome analyses across HBV genotypes and clinical cohorts. Third, translating mechanistic insights into therapies will require specific, cell-permeable inhibitors of NSUN2 or ALYREF, as well as tools for targeted epitranscriptomic editing (e.g., dCas13 fusions). Ultimately, validating the role of m^5^C in HBV pathogenesis will require robust in vivo models—including humanized liver mice and patient-derived organoids—alongside longitudinal patient cohort studies that link epitranscriptomic patterns to disease progression, treatment outcomes, and HCC risk.

In summary, m^5^C functions as both a facilitator of HBV replication and a suppressor of host immune defenses, placing it at the crossroads of viral persistence and oncogenesis. As our understanding of epitranscriptomic regulation deepens, m^5^C is poised to become not only a biomarker of HBV disease activity but also a novel therapeutic target. A multidisciplinary approach—integrating RNA biology, virology, immunology, and drug development—will be required to unlock the full potential of m^5^C-based interventions in chronic hepatitis B and HBV-related liver cancer.

## Figures and Tables

**Table 1:** Summary of major RNA modifications in viral infections.

Modifications	RNA Substrates	Typical Location	Key Roles in Viral Infections	References
m^5^C	mRNA, tRNA, rRNA, viral RNA	CDS/UTR	RNA stability, translation efficiency, immune evasion, nuclear export, and splicing	[10,11,12,13,14]
m^6^A	mRNA, lncRNA, circRNA, rRNA, viral RNA	3′ UTR, near stop codon	RNA stability, translation efficiency, immune evasion, nuclear export, splicing, and secondary structure	[15,16,17,18,19]
ac4C	mRNA, tRNA, rRNA, viral RNA	Coding region	RNA stability, translation efficiency, splicing, and secondary structure	[15,20,21,22,23,24,25]
Ψ	tRNA, rRNA, viral RNA	Structural regions	RNA stability, translation efficiency, and pre-mRNA processing	[26,27,28,29]
m^1^A	mRNA, tRNA, viral RNA	5′ UTR and coding regions	RNA stability and splicing	[22,23,30]
m^7^G	mRNA, rRNA, viral RNA	5′ cap, internal sites	RNA stability, translation efficiency, and ribosome biogenesis	[31,32,33,34]
Nm	rRNA, snRNA, mRNA, viral RNA	Ribose 2′-hydroxyl	RNA stability, translation efficiency, and pre-mRNA processing	[35,36,37]

**Table 2:** m^5^C in other viruses.

Virus	Writer	Notable m^5^C Sites	Key Functions	References
HIV-1	NSUN2	Gag-pol overlap, splice sites	Export, translation, splicing regulation	[136,142]
HCV	NSUN2	C7525 (NS5A)	RNA stability, replication, release	[141]
EV71	NSUN2	5′ UTR nt 584, CDS nt 1460	IRES-mediated translation, RNA stability	[11]
SARS-CoV-2	NSUN2	3′ UTR, ORF dynamics	RNA turnover, replication regulation	[140]
EBV	NSUN2	EBER1 C145	ncRNA stability modulation	[143]
MLV	NSUN2	5′ UTR, 3′ UTR	Genome packaging, RNA stability, immune evasion	[138]

## Data Availability

No new data were created or analyzed in this study. Data sharing is not applicable to this article.
